# Development of Specific Dosage Form: Wound Dressing—Current Progress and Novel Approaches

**DOI:** 10.3390/ph18111710

**Published:** 2025-11-11

**Authors:** Mihaela Violeta Ghica, Mădălina Georgiana Albu Kaya, Lăcrămioara Popa, Cristina Elena Dinu-Pîrvu

**Affiliations:** 1 Department of Physical and Colloidal Chemistry, Faculty of Pharmacy, Carol Davila University of Medicine and Pharmacy, 6 Traian Vuia Str., 020956 Bucharest, Romania; mihaela.ghica@umfcd.ro (M.V.G.); cristina.dinu@umfcd.ro (C.E.D.-P.); 2 Innovative Therapeutic Structures Research and Development Center (InnoTher), Carol Davila University of Medicine and Pharmacy, 6 Traian Vuia Str., 020956 Bucharest, Romania; 3 National Research and Development Institute for Textiles and Leather, Division of Leather and Footwear Research Institute, Department of Collagen, 93 Ion Minulescu Str., 031215 Bucharest, Romania

This Special Issue, ‘Development of Specific Dosage Form: Wound Dressing’, highlights the current progress in the development of modern wound dressings, indicating how dynamic and challenging this field remains. Wound healing is a multifactorial controlled process in which complications such as inflammation, pain, infection, delayed repair, and chronic injuries continue to represent meaningful obstacles for both patients and healthcare systems [1,2].

Globally, the burden of cutaneous skin lesions has received continuous interest in medical management, despite modern therapeutic progress. Skin wounds caused by trauma, surgery, burns, or underlying diseases (diabetes mellitus, chronic venous insufficiency, cancer) not only cause physical and psychological challenges to patients, but also significant socioeconomic costs [3,4]. Worldwide, current estimates suggest that more than 40 million individuals suffer from skin lesions (acute and chronic), and the financial impact of wound care is considerable, ranging from USD 28 billion to nearly USD 97 billion annually [5,6].

Wound repair is far from a linear process because it implicates a cascade of overlapping and interdependent phases requiring the coordinated action of numerous cellular and molecular entities [7]. This complexity explains why complications are common and why conventional dressings that just protect the surface of the wound are often insufficient. To address these limitations, research has been oriented toward advanced wound dressings that can actively accelerate the healing process [8]. Materials science, nanotechnology, and pharmaceutical science have contributed to the development of various systems, including hydrogels, sponges, hydrocolloids, films, foams, alginates, and textile-based platforms, loaded with different bioactive agents or nanoparticles [9,10]. These innovations not only aim to provide biocompatibility, but also antimicrobial protection, controlled drug release, and stimulation of tissue regeneration [11].

Therefore, the design of active wound dressings needs to address multiple requirements simultaneously: maintain a moist environment at the lesion site, protect against microbial contamination, promote angiogenesis and reepithelialization, enable the controlled release of bioactive agents, and ensure patient compliance and comfort [12,13,14,15,16].

Recent advances have expanded the use of various materials and the applications of different strategies to promote the healing process. Natural, semisynthetic, and synthetic biopolymers, nanostructured carriers, and hybrid composites are being explored in various pharmaceutical formulations [17,18,19,20]. Many of them are loaded with plant-derived compounds, anti-inflammatory, analgesic, and antimicrobial agents, growth factors, or nanoparticles, offering multifunctional behavior ranging from the protective role of the traditional dressings to the therapeutic role of promoting skin repair [21,22,23,24]. Additionally, the development of smart modern dressings sensitive to pH, temperature, and enzymatic activity is a promising objective with significant clinical applicability [25]. Despite this innovative reassuring progress, many challenges remain, including increasing the scale of the manufacturing process, regulatory authorization, costs, and the requirement for clinical approval [26].

In this Special Issue, eleven works (eight original research articles, two review articles, and one case report) are published, illustrating the comprehensive nature of the current strategies for developing modern wound dressings. These studies highlight how biomaterials and multifunctional and drug delivery systems influence wound management, providing both therapeutic effects and long-term perspectives for clinical applications.

Before considering the development of bioactive wound dressings, it is important to mention that one article in this Special Issue investigated the key role of polymers in designing wound dressings that can accelerate wound healing. Wong et al. developed a new crystalline glucose/mannose film as a modern polymeric wound dressing that combines non-adhesive behavior with moisture regulation, biocompatibility, and biodegradability (Contribution 1). This new formulation demonstrated a cellulose II-like crystalline model, with a smooth surface, suitable vapor permeability, and high water absorption, which are properties required for tissue healing. In vivo analysis on rats revealed that the glucose/mannose film substantially accelerated wound healing through collagen maturation and inflammation decline, promoting rapid epithelialization compared to the commercial alginate and hydrocolloid controls.

Four contributions included in this Special Issue investigated the integration of plant extracts as multifunctional bioactive agents, highlighting the therapeutic effects of natural products in wound healing management, along with polymeric supports.

In this regard, Alzahrani et al. investigated the protective effects of *Amaranthus graecizans* L. (AG), rich in polyphenols, vitamins, and minerals, against nonalcoholic fatty liver disease, induced by hyperlipidemia in rats (Contribution 2). Male Wistar rats received a high-fat diet and were treated with graded doses of AG solution (100, 200, or 500 mg/kg body weight) for two months. Phytochemical analysis indicated abundant phenolic compounds (especially chlorogenic and gallic acids) alongside a high content of fibers, potassium, calcium, and vitamins. AG supplementation significantly improved lipid profiles, reduced liver enzyme levels, and repaired the histological structure of liver tissues in a dose-dependent manner. Higher doses (500 mg/kg of AG) led to notable decreases in hepatic fatty accumulation and improved hepatocyte integrity.

Asiri et Venkatesan analyzed the therapeutic role of *Myristica fragrans* essential oil (MEO) in the wound healing process using a full-thickness excision wound model on Wistar rats (Contribution 3). GC-MS analysis indicated that MEO is a bioactive substance rich in many constituents (nitrobenzoate esters, cineole derivatives, and sesquiterpenoids) known for their antioxidant and anti-inflammatory properties. Topical application of 10% MEO significantly accelerated wound reduction, promoting complete epithelialization due to a decrease in pro-inflammatory cytokines and macrophage marker CD68 expression, as well as an improved antioxidant balance. Moreover, MEO modulated the apoptotic pathways by decreasing the activity of Caspase-3, supporting the durability of fibroblasts and the deposition of the extracellular matrix. Histological analysis also confirmed the enhanced deposition of collagen, reepithelialization, and reduced inflammatory infiltration.

Hasatsri et al. developed a new foam dressing based on gelatin and *Eclipta prostrata* leaf extract, with antibacterial and anti-inflammatory effects, aiming to combine traditional herbal activity with an advanced biomaterial design (Contribution 4). This new formulation exhibited a uniform porous structure, which was optimal to promote cell migration and proliferation. The foam dressing maintained a slightly acidic pH (~5), appropriate for antibacterial activity and skin tissue regeneration. Physicochemical tests indicated good absorption, dehydration, and flexibility properties, which are required for the treatment of injuries with low exudate levels.

Furthermore, another contribution investigated the combined therapeutic effects of different natural components (curcumin, resveratrol, and baicalin) on in vitro wound healing (Contribution 5). Jagiełło et al. investigated the wound healing potential of these three natural bioflavonoids by using fibroblast cell lines (Balb3t3 and L929) to evaluate their cytotoxicity (MTT assay) and migration (scratch test). None of the analyzed bioactive substances demonstrated cytotoxic effects, but they exhibited stimulatory effects on fibroblast proliferation and migration. By applying the Brian and Cousens mathematical model, the optimal concentration intervals to maximize cell viability and wound obstruction rate were established, providing quantitative support for optimizing dosage in designing new bioactive dressings.

Another study included in this Special Issue presents a combination of a plant-derived essential oil with a nonsteroidal anti-inflammatory drug, demonstrating their therapeutic effects in wound care. In line with this aspect, Plugariu et al. developed an innovative thermosensitive hydrogel based on polyurethane, proposed for the controlled transdermal release of meloxicam in order to solve the low solubility of this drug and its systemic side effects (Contribution 6). The novel formulation showed a temperature-responsive sol–gel transition with a self-healing ability and nanomicellar structure, which facilitates the sustained release of meloxicam. Moreover, the incorporation of *Origanum compactum* essential oil enhanced the antimicrobial activity and permeability of the drug.

Apart from the chemical compounds derived from different plants, some works included in this Special Issue focused on the development of wound dressings by incorporating various active pharmaceutical ingredients. These new formulations demonstrate how different bioactive agents can be effectively loaded into polymeric matrices to promote antimicrobial protection, reduce inflammation, and accelerate tissue regeneration.

Tudoroiu et al. developed smart-film dressings based on collagen and hydroxyethylcellulose, incorporating naproxen and phenol red for the dual purpose of therapy and real-time wound monitoring (Contribution 7). By combining natural and semisynthetic polymers with a nonsteroidal anti-inflammatory drug and a pH indicator, these new formulations are pH-responsive drug delivery systems, representing a clinically meaningful platform for real-time post-burn wound evaluation and localized treatment, combining diagnostic and therapeutic functions for modern wound healing management.

Albu Kaya et al. presented the design of biocomposite sponges based on collagen and hyaluronic acid and loaded with metronidazole, intended for localized treatment of periodontitis (Contribution 8). Structural characterization revealed the maintenance of collagen triple helix integrity and a porous structure, which enhances moisture uptake and the diffusion of the incorporated drug. This research represents a promising localized drug delivery system that manages infections in oral injuries, highlighting the significant potential of these bioactive polymeric biocomposites for wound and tissue regeneration.

Alongside the eight original research contributions included in this Special Issue, two comprehensive review papers are included that present recent advances and future directions and strategies in wound management.

The first review article, authored by Aldahish et al., presents the advancing potential of electrospun nanofibers based on silk fibroin nanofibers in developing bioactive dressings for diabetic wound healing (Contribution 9). The review illustrates an overview of the pathophysiological barriers associated with chronic diabetic wounds (oxidative stress, prolonged inflammation, and damaged angiogenesis) and discusses how silk fibroin-based nanofibers can surmount these limitations. This work describes the particular characteristics of silk fibroin—such as its biocompatibility, architectural resemblance to the extracellular matrix, and mechanical flexibility—which favor cellular proliferation, angiogenesis, and tissue remodeling. Moreover, the review also presents recent electrospinning technology that offers meaningful information about fiber morphology, porosity, or drug-loading ability, allowing for the development of bioactive wound dressings that induce the sustained release of therapeutic agents.

The second review paper, authored by Sharma et al., investigates the application of biodegradable electrospun scaffolds as modern materials intended for the regeneration of cutaneous lesions (Contribution 10). This paper describes how many types of biopolymers (natural and synthetic, including collagen, hyaluronic acid, chitosan, cellulose, polylactides, and polyhydroxyalkanoates) can be transformed into nanofibrous structures that can mimic the extracellular matrix, enhancing cell adhesion, proliferation, and skin tissue remodeling. The paper examines key electrospinning parameters, such as polymer concentration, viscosity, voltage, flow rate, and collector distance, as well as their influence on fiber structure, mechanical efficiency, and biological reactions. Moreover, it also highlights the potential of multifunctional nanofibers that can incorporate different therapeutic agents such as antimicrobial, anti-inflammatory, or growth-promoting compounds to accelerate wound healing.

The last contribution included in this Special Issue is a case report authored by Saberianpour et al. that presents the biological interactions between the components of wound exudate and dressing biomaterials, discussing how they influence healing outcomes and establishing the potential biomarkers of wound progression (Contribution 11). Six patients with acute surgical wounds were treated using two general dressings: Atrauman (a hydrophobic polyester-based tulle dressing) and Melolin (a hydrophilic hydrocolloid dressing). Cellular and biochemical analyses indicated that efficient healing correlated with the retention of progenitor (CD105^+^) and M2 macrophage cells on the Atrauman interface, while the absorption of these cells into Melolin was linked to the slower or impaired recovery. Moreover, Melolin adsorbed a broader range of inflammatory proteins, whereas Atrauman primarily bound albumin, limiting inflammatory activation.

Together, the original articles, reviews, and the case report collected in this Special Issue collectively form an overview of the rapid progress and persistent challenges in the development of wound dressing. Collectively, they demonstrate how innovative biomaterials, natural compounds, and active pharmaceutical ingredients can be combined to create dressings that not only protect, but also actively stimulate tissue repair. From plant-derived compounds and smart polymeric matrices to drug-loaded hydrogels and multifunctional nanofibers, these contributions illustrate the union of material science, pharmacology, and biotechnology in modern wound management. The reviews further consolidate current knowledge and highlight electrospinning, nanostructuring, and biopolymer engineering as transformative approaches for next-generation dressings. The included case report emphasizes the growing importance of understanding biological interactions between wound exudates and dressing materials to optimizing clinical outcomes. Altogether, this Special Issue indicates a field evolving toward personalized, responsive, and multifunctional wound care systems that are capable of accelerating healing while improving patient comfort and quality of life.
